# Spatial registration of serial microscopic brain images to three-dimensional reference atlases with the QuickNII tool

**DOI:** 10.1371/journal.pone.0216796

**Published:** 2019-05-29

**Authors:** Maja A. Puchades, Gergely Csucs, Debora Ledergerber, Trygve B. Leergaard, Jan G. Bjaalie

**Affiliations:** 1 Neural Systems Laboratory, Department of Molecular Medicine, Institute of Basic Medical Sciences, University of Oslo, Norway; 2 Kavli Institute for Systems Neuroscience, Norwegian University of Science and Technology, NTNU, Norway; Universidad de Salamanca, SPAIN

## Abstract

Modern high throughput brain wide profiling techniques for cells and their morphology, connectivity, and other properties, make the use of reference atlases with 3D coordinate frameworks essential. However, anatomical location of observations made in microscopic sectional images from rodent brains is typically determined by comparison with 2D anatomical reference atlases. A major challenge in this regard is that microscopic sections often are cut with orientations deviating from the standard planes used in the reference atlases, resulting in inaccuracies and a need for tedious correction steps. Overall, efficient tools for registration of large series of section images to reference atlases are currently not widely available. Here we present QuickNII, a stand-alone software tool for semi-automated affine spatial registration of sectional image data to a 3D reference atlas coordinate framework. A key feature in the tool is the capability to generate user defined cut planes through the reference atlas, matching the orientation of the cut plane of the sectional image data. The reference atlas is transformed to match anatomical landmarks in the corresponding experimental images. In this way, the spatial relationship between experimental image and atlas is defined, without introducing distortions in the original experimental images. Following anchoring of a limited number of sections containing key landmarks, transformations are propagated across the entire series of sectional images to reduce the amount of manual steps required. By having coordinates assigned to the experimental images, further analysis of the distribution of features extracted from the images is greatly facilitated.

## Introduction

Interpretation of brain-related data requires precise information about the anatomical location from which the data are derived. Recent advancements in the availability and usability of digital three-dimensional (3D) atlases and associated tools have made it feasible to associate many different data types to these known standards [1–3]. This in turn has given the research community the ability to greatly expand the capability to perform powerful and unique analyses with the option of looking beyond single studies or data modality.

While the ability to register single two-dimensional (2D) images to 3D atlas slices exists in a number of tools [4–7], many are coding based interfaces and therefore not user friendly to the wider neuroscience community. In addition, the ability to treat the atlas as a true 3D image is not always feasible. Most paper, and even 3D atlases, can only be viewed in the standard coronal, sagittal, and horizontal planes, with few tools allowing views in non-standard planes. This greatly limits the ability to match real life data to the high quality datasets in canonical atlases.

**Fig 1:**
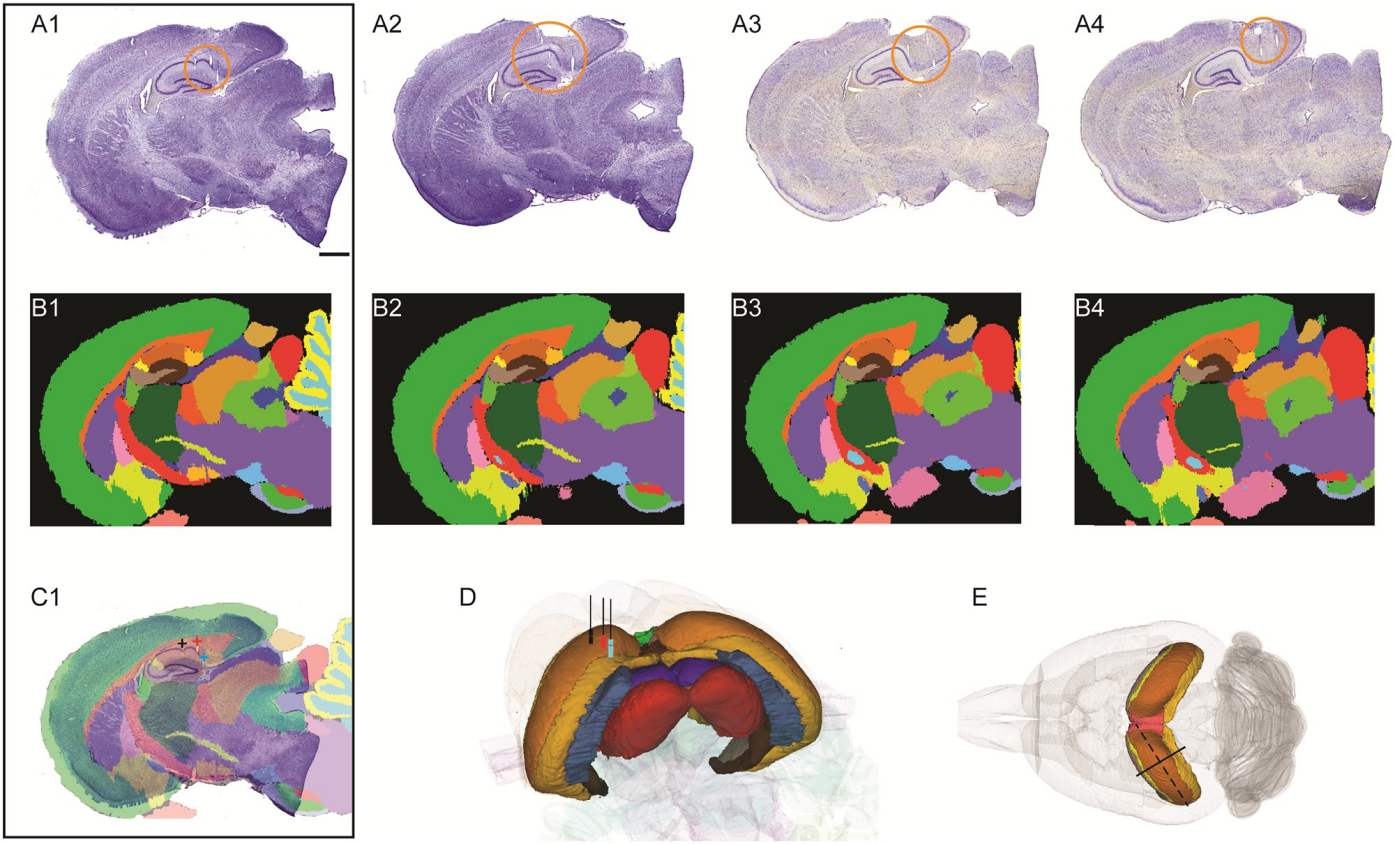
Mapping electrophysiological recording sites in the rat hippocampal formation. (A1-A4) Raw images of thionin-stained histological sections cut perpendicularly to the long axis of the hippocampus in order to visualize electrode positions (illustrated in E). (B1-B4) Corresponding customised atlas maps from the rat Waxholm Space atlas, obtained from the QuickNII tool after registration. (C1) Visualisation of overlayed images A1 and B1 in QuickNII, allowing to read and collect coordinates for electrode tracts positions. The name of the region appears when the mouse is pointed to that region. (D) The coordinates can be plotted and visualized in a 3D viewer (e.g. MeshView on https://www.nitrc.org/projects/meshview) enabling comparisons between animals or different experiments. (E) 3D visualization of the rat hippocampal formation, which is a C-shaped structure curving obliquely from the midline towards the temporal lobe of the brain. (Scale bar, 1 mm).

The importance of being able to examine an atlas off-axis is illustrated in Fig 1. Here the angle is oblique; perpendicular to the long axis of the rat hippocampus about halfway between the coronal and sagittal planes (Fig 1A1–1A4). The section angle was chosen to identify the precise locations of a series of electrode tracks, but the anatomical features are very difficult to interpret, even for experts. Generating custom atlas plates that match the image orientation provide the foundation necessary to properly identify anatomy (Fig 1B1–1B4). This example demonstrates that the ability to treat an atlas as truly 3D and generate customized reference atlas plates is an important feature for a registration tool. Further, when section angles are aimed at the standard planes, the matching of section images to canonical atlases is challenged by the often-occurring small, but non-trivial, deviations from the intended angle of orientation.

As the community moves towards analyzing brains in 3D, there has been a push towards collecting datasets that span the full brain, especially in the rodent [1, 2, 8–13]. While some laboratories have developed tools and workflows to reconstruct image series covering the whole brain back into 3D and register them to a canonical atlas [1, 11, 14–16]; the majority of investigators do not have access to these kinds of technical resources. Registering an individual 2D image to a 3D atlas is challenging and time-consuming, and registering a large series, especially when a number of slices lack anatomical information, is nearly impossible. However, if individual images in a series can be treated as pieces of a puzzle, key landmarks in some slices can be used to aid the placement of slices lacking such information.

We have created a new neuroinformatics tool called QuickNII (https://www.nitrc.org/projects/quicknii), specifically to aid the process of bringing a series of 2D images into 3D space in register with a reference atlas. QuickNII is a standalone, GUI-based, open access tool tailored for efficient assignation of spatial location to serial microscopic brain images.

Two versions are available; one for the Waxholm Space rat brain atlas [3, 17] (available from https://www.nitrc.org/projects/quicknii and one for the Allen Brain Mouse Atlas [1, 2] (available from http://www.brain-map.org/). Within QuickNII, these volumetric brain references are used to generate customized atlas plates that match the spatial orientation of any experimental sections.

## Results and discussions

### Registration of serial sections images to a reference atlas with QuickNII

To avoid the technical and scientific challenges related to adjusting and distorting very large images, QuickNII linearly stretches the atlas plates to match down-sized experimental section images. This establishes the spatial relationship between the atlas and input image necessary to assign spatial coordinates to the image. Anatomical location is defined by superimposing customized atlas images onto section images in a process we call “anchoring.”

**Fig 2:**
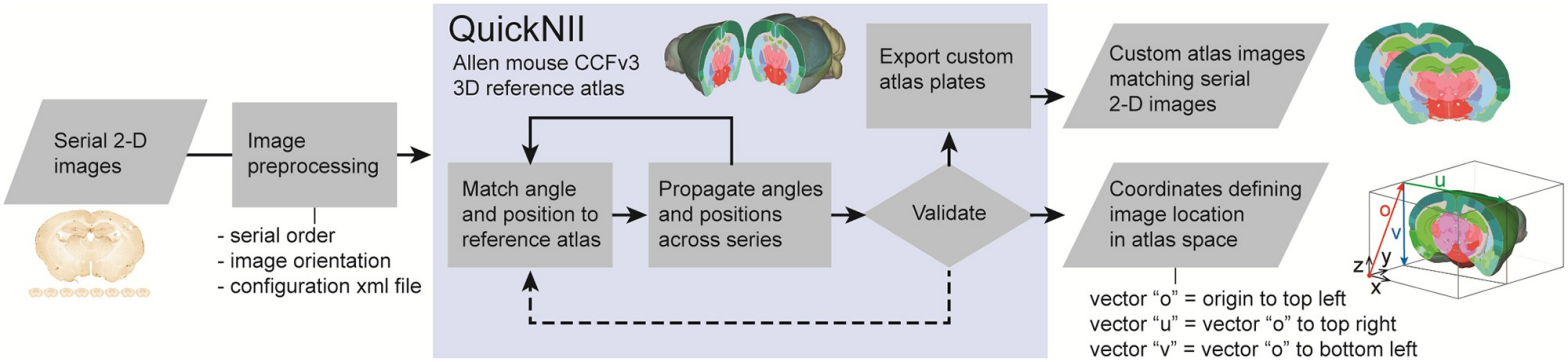
QuickNII workflow. Diagram showing key steps of the workflow used to anchor serial section images to the Allen Mouse CCF v.3 reference atlas space using QuickNII. Following initial preprocessing steps where the sequence and orientation of the serial images is validated and a configuration XML file is generated, images are imported to QuickNII. The user will use visible landmarks in the experimental image to manipulate the atlas, adjusting position, scale, and orientation (rotation and tilt) that best matches the selected image. QuickNII automatically propagates information about position, scale, and tilt to the entire series. By iterative anchoring of selected key sections, the user can optimize the automatically propagated parameters. A final validation for each image is strongly recommended. Output from QuickNII is a series of custom atlas maps matching each anchored experimental image, and an XML file describing a set of vectors (*o*, *u*, and *v*) that define the position of each image relative to the technical origin of the reference atlas used.

The high-level workflow of how one uses QuickNII to register a series of images and obtain the spatial information for the images is shown in Fig 2 and explained in detail in the User guide and on https://www.nitrc.org/projects/quicknii.

**Fig 3:**
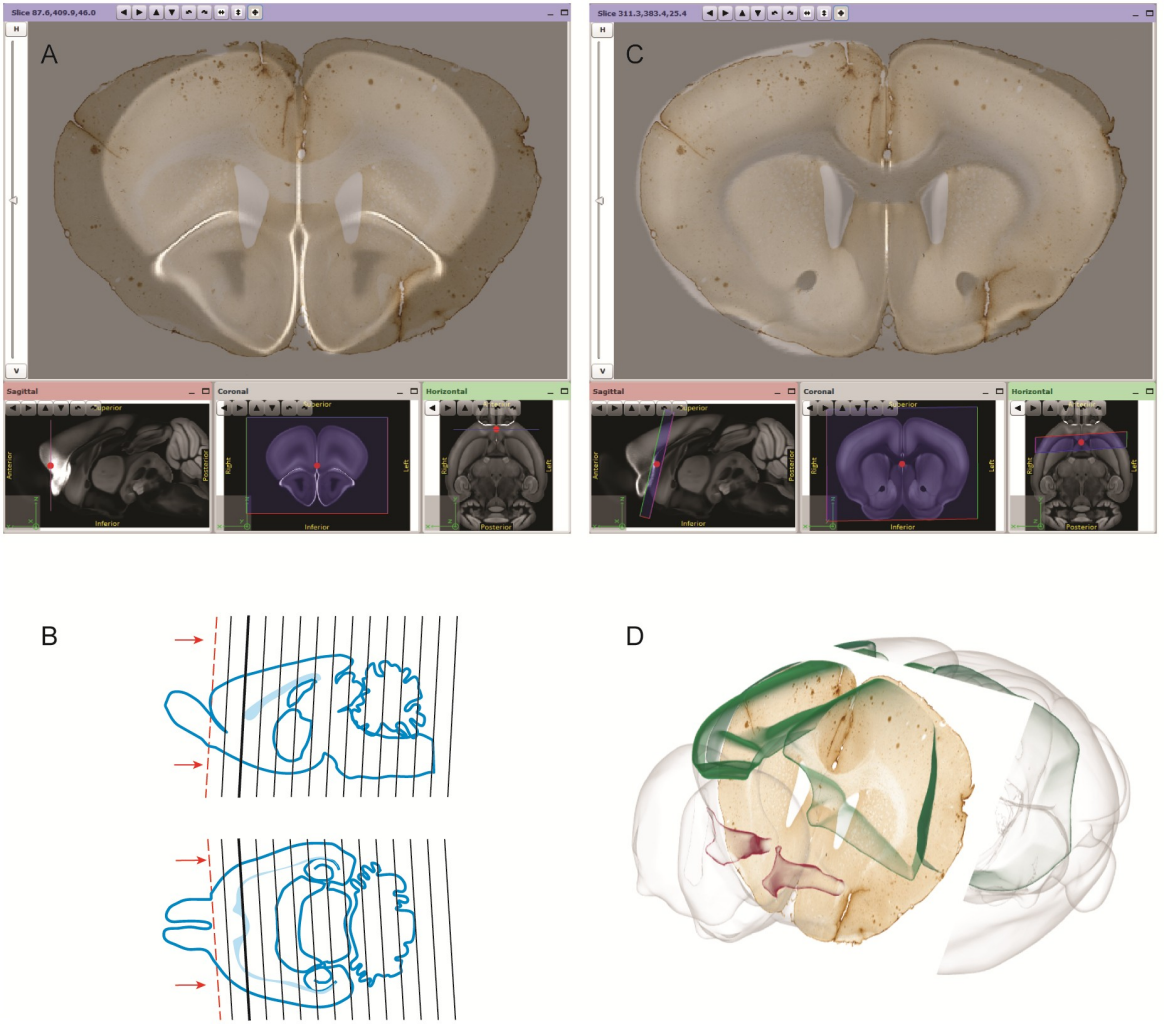
Registration of a brain section image to the Allen mouse reference atlas with QuickNII. (A) Before registration in the QuickNII interface, the section image and the reference atlas are shown in a default Bregma position with the dorsoventral (DV) and mediolateral (ML) angles at position 0. (B) Line drawing of the reference atlas plate matching the location and tilted to fit the DV and ML angles of orientation of the section shown in A. (C) After registration in QuickNII, Bregma position, DV angle (+13) and MV angle (-4) have been adjusted by the user. Registration of more than one section to atlas results in repositioning of the other sections in the series relative to the reference atlas. Iterative manual registration of sections is performed until a satisfactory result is reached across the entire series of images. (D) The section images anchored with QuickNII can be visualized in a 3D viewer tool (scalablebrainatlas.incf.org), where atlas meshes can be selected and viewed (here the anterior commissure in red and the somatosensory and somatomotor areas in green).

The principal steps are:

Step 1: Users start by sectioning and collecting contiguous brain tissue slices. They typically end up with a series, or multiple series of slices with different stains or types of expression on alternating slices.
Step 2: The slices are digitized and the images organized sequentially before pre-processing to ensure proper orientation of all images, eventual renaming and downscaling (see Methods). Upon completion, the user should have a series of image files consistent with the original brain slices.
Step 3: Using the program “FileBuilder”, provided together with QuickNII, the user generates an XML descriptor file (see Methods), and QuickNII uses this information to generate an initial distribution of the images in the chosen atlas (S1 Fig, situation 1). Thus, when the user begins, the slices are sorted in the correct order and may already be roughly located in the appropriate position, giving a first approximation that can be fine-tuned by the user. Working in QuickNII gives the ability to view an image superimposed on the atlas modality of choice (multiple data modality options for each atlas) with varying levels of opacity in relation to the atlas (Fig 3A).
Step 4: The user can browse through the images and identify landmarks for accurate positioning of each slice by adjusting the dorso-ventral and the mediolateral angles (Fig 3B). The location of the slice can be viewed in all three commonly used planes. This along with the power to view and adjust off-axis cuts through the atlas enables a better fit. Once the slice is positioned in the atlas, the atlas image can be scaled horizontally or vertically to match the size of the image slice. When the user is satisfied with the match between an image and the reference atlas, they can anchor it to the atlas (Fig 3C). The approach used here differs from other published methods where the anteroposterior position of the section in the atlas is only approximated and non-linear deformations methods are applied directly, without taking into account the section angle of orientation [6, 18].
Step 5: Once an image in a series has been anchored to the atlas, a new spacing distribution is automatically calculated for the remaining images. The spacing continues to be recalculated as additional images are registered (S1 Fig, situation 3). A major advantage of this method is that users can select images with more identifiable anatomical landmarks to map to the atlas, and in turn, this information is used to place the other slices without obvious landmarks.
Step 6: Once two images near the start and the end of the series have been anchored to the atlas by the user, the remaining sections should be in close proximity to their appropriate position in the atlas. At this point, the user can rapidly step through each image in the series and fine-tune its position. The result of this procedure is considered to be a global anchoring, aimed at the best possible matching of the atlas to the brain region present in the experimental section. A local anchoring approach can be chosen if the user wishes to achieve the best possible match to one or a few brain regions, disregarding neighboring regions.
Step 7: The user saves a new XML file, in which the spatial coordinates of each 2D image in relation to the atlas is captured.

### QuickNII output: Examples of use

#### Location and visualization of electrode tracts in the rat Waxholm Space Atlas

In QuickNII, the coordinates of any location in a 2D image are displayed when pointing with the mouse cursor (See User guide). The coordinates can be manually collected and plotted in the 3D viewer, MeshView (Fig 1D), enabling analysis of spatial distributions and comparisons of the distribution of selected features across animals.

Depending on the quality of the histological material and the type of analysis to be pursued, an additional non-linear adjustment of the registration between the atlas slice and section image may be required.

#### Visualization of mouse brain sections in the Allen mouse brain atlas space

For image series anchored to the Allen mouse brain atlas, users can visualize their sections together with atlas meshes in the Scalable Brain Atlas Composer tool [19] in an interactive manner (Fig 3D). This viewer allows uploading directly the output from QuickNII, i.e. the output XML file and png images.

### Opportunities for interoperable use of neuroscience data

Recent international efforts in large scale integration of neuroscience data and cellular and cell type profiling, such as the European Union Human Brain Project (http://www.humanbrainproject.eu/en/), Human Cell Atlas (http://www.humancellatlas.org/), and NIH BRAIN Initiative Cell Census Network (http://www.biccn.org), recognize the importance of using 3D common coordinate frameworks and the need for developing the relevant computational tools and infrastructures enabling heavy computing power; not available for single research groups.

**Fig 4:**
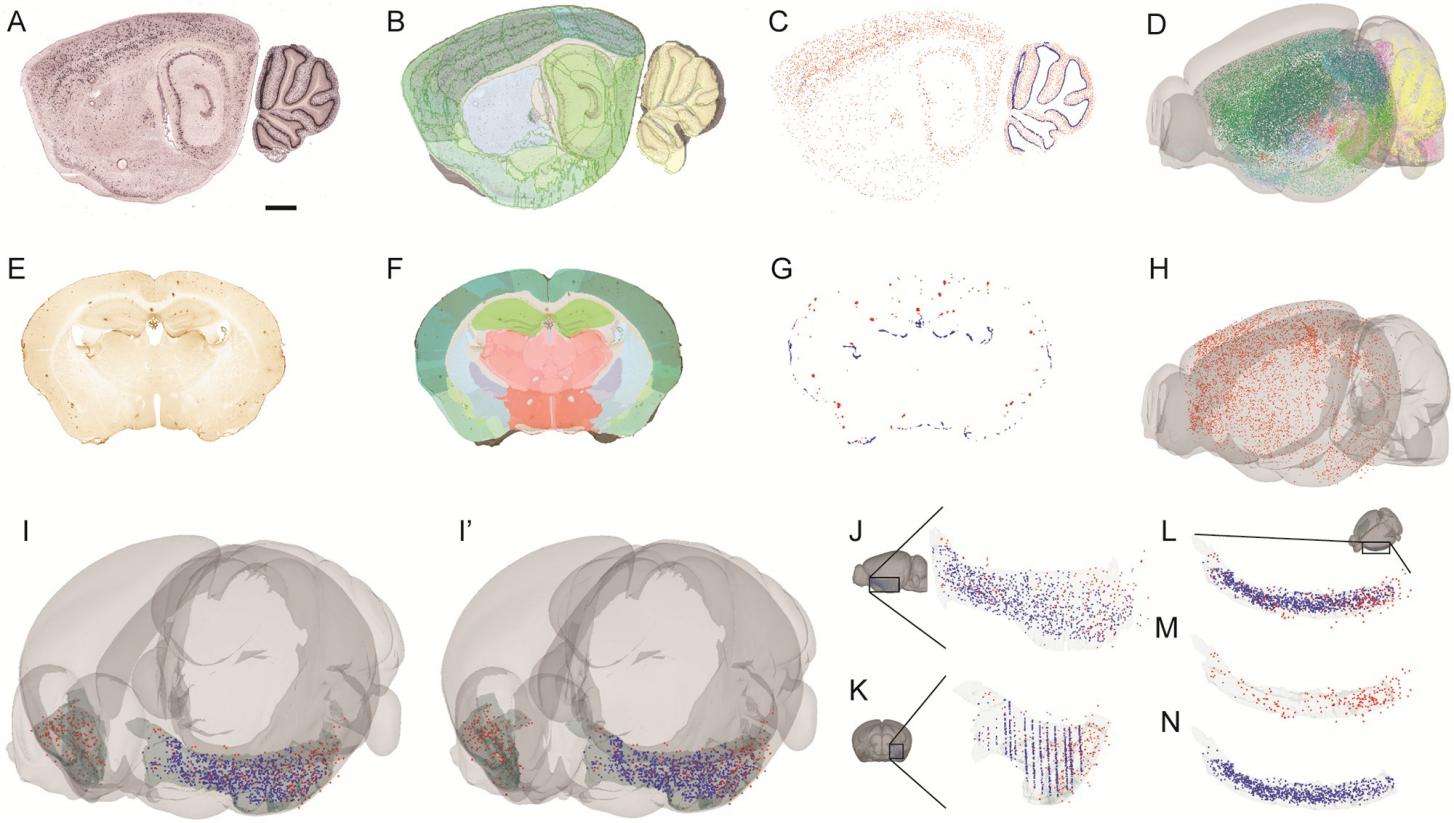
Spatial integration of data from different experimental image series. The figure illustrates how histological features from differently oriented histological images can be extracted and compared after spatial registration to a common reference atlas (Allen Mouse Brain Atlas). (A) Shows one sagittal microscopic image from a series of a mouse brain, labelled by in-situ hybridization with an RNA probe against parvalbulmin (data from mouse.brain-map.org/, case 19056). (E) Shows one coronal microscopic image from a series of a transgenic mouse brain (the Tg2576 model for Alzheimer disease) in which amyloid plaque has been visualized using immunohistochemistry. (B, F) Using QuickNII, the two series of sections are registered to the Allen Mouse Brain Atlas. The previous sections are shown with their respective atlas maps superimposed. (C, G) Labelled features of interest are extracted using the machine learning software tool ilastik (ilastik.org). (D, H) 3D visualization of the extracted features which, represented by centroid point coordinates, are displayed in 3D atlas space. Data points in (D) are color coded by regions in the atlas. (I-I’) Stereo pair images illustrating how data points from the two experimental data sets (blue dots, parvalbumin positive cells; red dots, amyloid plaques) can be co-visualized in an atlas region of interest, in this example the piriform cortex. To see the stereo images the viewer must cross the eye axis to let the pair of images merge into a 3D image. (J-N) Images illustrating combined data points in the piriform cortex in views from lateral (J), anterior (K) or obliquely from dorsal, perpendicular to the cortical sheet (L-M), as indicated on the inset brain images. The red and blue data points are displayed together (L) and separately (M, N). Scale bar, 1 mm.

In QuickNII, while anchoring is performed on downsized images; the full size images can inherit atlas-related information about brain structure (see Methods). Exporting the coordinates generated by QuickNII in XML format allows the option for further analysis and viewing in other software tools. In this context, we have developed an image viewer tool embedded in a web application that reads QuickNII XML files, displays high-resolution microscopic images (Microsoft Deep Zoom file format) with atlas overlay images, and provides basic annotation functionalities. This tool, which requires the possibility of accessing pyramid versions of high-resolution images, is used to inspect microscopic details and record points of interest as X,Y,Z coordinates in reference atlas space. These derived coordinates can be visualized as geometric objects together with meshes representing anatomical structures derived from a reference atlas. In this way, datasets from different experiments can be integrated in the same reference space, allowing quantitative analyses in response to diverse neuroanatomic-related questions. Examples of applications include the ability to accurately map recording electrode locations in 3D space and identify the areas and regions in which they were located (Fig 1), as well as the ability to co-localize tracer injection sites, multiple categories of neurons, or other labeled features in the brain, as illustrated in Fig 4. By combining the atlas maps with segmented images (Fig 4C and 4G), the user will be able to extract spatial coordinates of objects and compare data across datasets as illustrated in Fig 4I–4N. This workflow, albeit not accessible to individual neuroscience laboratories is part of an infrastructure created in the Human Brain Project with the aim to make it available to the whole community [20].

## Conclusion

We have here presented a strategy, workflow, and user-friendly tool for rapid registration of many microscopic 2D images to 3D atlas space. QuickNII gives researchers a tool to fairly quickly and easily locate series of 2D image slices in the 3D space of canonical atlases. We have used QuickNII for mouse and rat datasets and developed well-defined procedures and tutorials for anchoring 2D image series. These procedures together with training material have been successfully used by several test site laboratories [21] and in several Human Brain Project training workshops. QuickNII with its tutorial is available via the NITRC homepage (https://www.nitrc.org/projects/quicknii).

## Materials and methods

### Histological material

The material shown in Fig 1 is from a 5 months male Long Evans rat, from which histological sections were stained with Nissl. The experiments related to this animal were performed in accordance with the Norwegian Animal Welfare Act and the European Convention for the Protection of Vertebrate Animals used for Experimental and Other Scientific Purposes, and in compliance with protocols approved by the Norwegian Animal Research Authorities, permit number 7163. High-resolution section images were acquired using an automated slide scanner system (Axio Scan, Carl Zeiss MicroImaging, Jena, Germany). The material shown in Figs 2, 3 and 4(E) and 4(F) is from an 18 months male mouse model (Tg2576 mouse) for Alzheimer disease, provided by M. Hartlage-Rübsamen and S. Rossner. Coronal sections were stained for amyloid plaques (4G8 antibody, Signet Lab Cat# 4G8, RRID:AB_2313891) and visualized by immunohistochemistry. The animal experiments were approved by Landesdirektion Sachsen, license no T28/16. High-resolution section images were acquired using an automated slide scanner system (Axio Scan, Carl Zeiss MicroImaging, Jena, Germany). The material shown in Fig 4(A, B) is from a 57BL/6J, P56 male mouse. It demonstrates distribution of parvalbumin mRNA in sagittal brain sections identified using in-situ hybridization. The section images were provided by the Allen Institute for Brain Science, Parvalbumin in situ hybridization, experiment no. 2007 75457579, 2007, available from: http://mouse.brainmap.org/experiment/show/75457579 (accessed 28 November 2017).

### Preparation of image data

#### Preprocessing of images

QuickNII v2.2 supports standard web-compatible image formats, 24-bit PNG and JPEG. Images can be loaded up to the resolution of 16 megapixels (e.g. 4000x4000 or 5000x3000 pixels), however QuickNII does not benefit from image resolutions exceeding the resolution of the monitor in use. For a standard FullHD or WUXGA display (1920x1080 or 1920x1200 pixels) the useful image area is approximately 1500x1000 pixels, using a similar resolution ensures optimal image-loading performance.

Preprocessing of images (downsampling, rotation, renaming) can be achieved with open access software tools (e.g. ImageMagick, Matlab scripts) or python scripts found in many open source libraries (e.g. PIL) to fulfill these requirements (converting to PNG or JPEG and downscaling to screen-like size), but QuickNII allows storage of original image dimensions as part of its series descriptor. Serial section images should be assigned consecutive serial numbers, preferably indicated by three-digit numbers at the end of the file name, e.g. Sample_ID_s001.tif. Section sampling is given by the serial numbers. The section images are collected in a folder.

#### Creation of the image series descriptor file

The program “FileBuilder.bat” is provided with QuickNII. This program asks for the folder where your images are located, reviews the image files, and generates an xml file. This file can be saved and serves as input file for the given collection of images in QuickNII. FileBuilder uses numbers in the file names in order to generate the serial ordering. If section numbers are not recognized, the user will have the option to number the images in the FileBuilder program.

### Software technical description

The detailed descriptions can be found on the QuickNII page on https://www.nitrc.org/projects/quicknii (RRID:SCR_016854).

### Coordinate system

QuickNII uses the Neuroimaging Informatics Technology Initiative (NIfTI) coordinate system (https://nifti.nimh.nih.gov/) for the reference atlases. Coordinates in NIfTI-space are expressed in voxels. The origin is the bottom left corner of the most anterior coronal plane, x axis increases from left to right, y axis increases from posterior to anterior, and the z axis increases from inferior to superior. As 2D images do not contain spatial information in relation to real space, they are described in the reference atlas using “anchor vectors”. The 2D images are not modified during this process; instead, each image is described by *the corresponding atlas slice* in the reference atlas and specified with the following vectors:

- ***o*** points from the technical origin to the top left corner of the given atlas slice, essentially specifying an origin for that atlas slice.
- ***u*** points from ***o*** to the top right corner of the atlas slice, specifying the horizontal edge of the atlas slice.
- ***v*** points from ***o*** to the bottom left corner of the atlas slice, specifying the vertical edge of the atlas slice.

### Correspondence between image and atlas coordinates

As ***o***, ***u*** and ***v*** vectors are expressed in voxels, they represent a simple and direct connection between image pixels and atlas voxels, a weighted sum of ***u*** and ***v*** vectors has to be added to the origin, ***o***. ***o*** itself is the top-left corner (0,0 pixel coordinate) of the image, ***o***+***u*** is the top-right corner (width-in-pixels,0), ***o***+***v*** is the bottom-left corner (0,height-in-pixels), ***o***+***u***+***v*** is the bottom-right corner (width-in-pixels,height-in-pixels), and everything else is somewhere in between, weights of u and v running from 0 to 1 (See Table 1). Practically x and y pixel coordinates have to be divided by width-in-pixels and height-in-pixels respectively.

**Table 1:** Table showing the corner cases and calculation for an arbitrary pixel (where x,y denote pixel coordinates and w,h abbreviates width-in-pixels and height-in-pixels).

	Vector	Coordinates
Top-left corner	***o***	[o_x_ o_y_ o_z_]
Top-right corner	***o***+***u***	[o_x_+u_x_ o_y_+u_y_ o_z_+u_z_]
Bottom-left corner	***o***+***v***	[o_x_+v_x_ o_y_+v_y_ o_z_+v_z_]
Bottom-right corner	***o***+***u***+***v***	[o_x_+u_x_+v_x_ o_y_+u_y_+v_y_ o_z_+u_z_+v_z_]
Any pixel	***o***+x/w*u+y/h*v	[o_x_+x/w*u_x_+y/h*v_x_ o_y_+x/w*u_y_+y/h*v_y_ o_z_+x/w*u_z_+y/h*v_z_]

The vectors can also be used to build a transformation matrix:

$$\left(\begin{array}{ccc} x_{v} & y_{v} & z_{v} \end{array})\;=\;(\begin{array}{ccc} x_{p}/w & y_{p}/h & 1 \end{array})*[\begin{array}{ccc} u_{x} & u_{y} & u_{z}\\ v_{x} & v_{y} & v_{z}\\ o_{x} & o_{y} & o_{z} \end{array}\right]$$

Where x_v_, y_v_, z_v_ are coordinates in atlas voxels, for x_p_, y_p_ image pixels. w and h again abbreviates image dimensions, width-in-pixels and height-in-pixels.

### Correspondence between atlas coordinates in voxels and physical space

#### Transformation of mouse voxel coordinates to Allen CCFv3

(http://help.brain-map.org/display/mousebrain/API):

$$\left(\begin{array}{cccc} x_{a} & y_{a} & z_{a} & 1 \end{array})\;=\;(\begin{array}{cccc} x_{v} & y_{v} & z_{v} & 1 \end{array})*[\begin{array}{cccc} 0 & 0 & 25 & 0\\ -25 & 0 & 0 & 0\\ 0 & -25 & 0 & 0\\ 13175 & 7975 & 0 & 1 \end{array}\right]$$

### Transformation of rat voxel coordinates to Waxholm space

$$\left(\begin{array}{cccc} x_{w} & y_{w} & z_{w} & 1 \end{array})\;=\;(\begin{array}{cccc} x_{v} & y_{v} & z_{v} & 1 \end{array})*[\begin{array}{cccc} 0.0390625 & 0 & 0 & 0\\ 0 & 0.0390625 & 0 & 0\\ 0 & 0 & 0.0390625 & 0\\ -9.53125 & -24.3359375 & -9.6875 & 1 \end{array}\right]$$

Where x_v_, y_v_, z_v_ are coordinates in atlas voxels (RAS axis orientation and order), x_a_, y_a_, z_a_ are coordinates in Allen CCFv3 (PIR axis orientation and order, values are expressed in μm-s) and x_w_, y_w_, z_w_ are coordinates in Waxholm Space (RAS axis orientation and order, values are expressed in mm-s).

### Output data

After the anchoring procedure, the images are linked to the 3D reference atlas. The output consists of a new XML file where the vectors describing the corresponding atlas slice are stored for each image file in the series.

### Example of XML descriptor extended with anchoring vectors

<?xml version = '1.0' encoding = ’UTF-8'?>

<series name = ’Test series'>

<slice filename = ’sampleID_s002.png’ nr = '2' width = '24723' height = '18561'

anchoring = ’ox = 312.2&oy = 533.8&oz = 218.4&ux = -185.7&uy = -35.5&uz = 6.6&vx = -4.6&vy = -7.5&vz = -171.4'/>

<slice filename = sampleID _s008.png’ nr = '8' width = '24722' height = '17507'

anchoring = ’ox = 334.82142136461607&oy = 485.7990978550188&oz = 251.62087421842926&ux = -228.65532680537657&uy = -13.31692466388239&uz = -11.98107468791568&vx = 11.021383786310935&vy = -7.15410850678 6784&vz = -202.38817266644594'/>

</series>

slice.anchoring: x-y-z coordinates of o-u-v vectors in URL-encoded format.

While anchoring vectors are available for all section images all the time (see section on ‘Propagation’ below), the XML descriptor contains only the ones approved by users via pressing the ‘Store’ button. This approach encapsulates the distinction between anchor vectors being ‘estimated’ (algorithmically) or ‘verified’ (by the user), and conserves storage space at the same time.

### Same example as JSON descriptor

{"name":"Test series","slices":[

{"nr":2,"filename":"sampleID_s002.png","width":24723,"height":18561,

"anchoring":[312.2,533.8,218.4,-185.7,-35.5,6.6,-4.6,-7.5,-171.4]},

{"nr":8,"filename":"sampleID_s008.png","width":24722,"height":17507,

"anchoring":[334.82142136461607,485.7990978550188,251.62087421842932,-228.6553268,-13.316924663882391,-11.981074687915681,11.021383786310937,-7.15410850678,-202.3881726664459]}

]}

The field “anchoring” in the JSON format is a compact list of the 9 anchor components, in o_x_, o_y_, o_z_, u_x_, u_y_, u_z_, v_x_, v_y_, v_z_ order.

### Export atlas slices

"Export Slices" generates atlas slices for all section images in the series. The images are generated into a timestamped subfolder next to the series, "Slices-YYYYMMDDHHmmSS" format. All available templates (MRI, DTI, pMRI, Nissl) are sliced as well as the segmentation volume. The slices follow the resolution of the atlas and thus they are typically much smaller and have a different aspect ratio than the original images. QuickNII works with the exact same slices and stretches them over the actual images to achieve the view what the user sees: all four corners of the slice are positioned at the corners of the image. This can be reproduced with arbitrary image manipulation software. A progress bar is showing the generation of slices, QuickNII cannot be used for anything else until the process is finished.

Custom atlas slices are generated in the native resolution of the atlas (i.e. 1 pixel is a 40x40 um2 square for WHS Rat and 25x25 um2 square for Allen Mouse), and thus they are independent from the resolution of the original images and usually much smaller.

Scaling along horizontal and vertical axes is done separately, the scaling factors are expected to be close to each other, but they are not necessarily equal.

Let w,h denote the width and height of the original image in pixels, and c_w_,c_h_ denote the width and height of the custom atlas slice.

In order to get corresponding c_x_,c_y_ position in the customized atlas slice for any x,y position in the original image, the following calculation can be done:

$${\text{c}}_{\text{x}}=\text{x}*{\text{c}}_{\text{w}}/\text{w}$$

$${\text{c}}_{\text{y}}{=\text{y}\;\text{c}}_{\text{h}}\text{/h}$$

where x = 0…(w-1), y = 0…(h-1) and the resulting c_x_ = 0…(c_w_-1), c_y_ = 0…(c_h_-1), bounds are inclusive.

With matrix notation:

$$\left(\begin{array}{cc} c_{x} & c_{y} \end{array})\;=\;(\begin{array}{cc} x & y \end{array})*[\begin{array}{cc} \frac{c_{w}}{w} & 0\\ 0 & \frac{c_{h}}{h} \end{array}\right]$$

### Export formats

- for templates and segmentation: 24-bit (truecolor) PNG files, "<original filename>-<template or segmentation name>.png" naming pattern is used
- for segmentation slices a "<original filename>-<segmentation name>.flat" file is generated too, which is an uncompressed binary format containing atlas identifiers
- palette: "<segmentation name>.json" file contains colors and names for segmentation.

".flat" format:

Offset 0 [byte]: Bpp, Bytes per pixel (1 or 2 in current uses) Offset 1 [32-bit integer]: width (in pixels) Offset 5 [32-bit integer]: height (in pixels) Offset 9: Bpp*width*height bytes of pixel (atlas identifier) data.

Quantities are stored in big-endian order.

Palette format:

JSON file containing an array of (index, red, green, blue, name) tuples, where name is string the rest are numbers. Index is deliberately redundant; it contains the actual array index.

### System requirements and software availability

System requirements for the QuickNII tool are

- Microsoft Windows: 64-bit operating system, Windows 7 or later
- Apple macOS: OS X 10.9 (Mavericks) or later
- 3 gigabytes RAM
- Display resolution minimum 1440 pixels wide and minimum 650 pixels high.

The QuickNII software is available for download from https://www.nitrc.org/projects/quicknii.

## Supporting information

S1 Fig Illustration of DV and ML angle adjustments. (A) Line drawing of an experimental rat brain section cut at an angle slightly tilted compared to the standard coronal plane. The section is one in a series of sections through the brain. Through the registration process in QuickNII, correctly angled and positioned atlas plates from a 3D rat brain reference atlas (Waxholm Space rat brain atlas) will be superimposed onto each section in the series. (B) Line drawing of a coronal reference atlas plate, found to be close to the section shown in A. The slight difference in angle of orientation between the images in A and B is most easily observed in the hippocampal region, marked with an asterisk. (C) Line drawing of a reference atlas plate matching the location and tilted to fit the angle of orientation of the section shown in A. In the middle and bottom rows, the assumed position of the section shown as is indicated in situation 1 (solid line relative to sagittal and horizontal atlas diagrams, shown in the middle and lower rows, respectively), whereas the angle of orientation of the atlas plate shown in C is indicated in situation 2. The angle of orientation and the scaling (not shown) defined for the adjusted atlas plate is automatically propagated across the series, while distances among sections are automatically stretched or compressed. (D) Section from A (grey) and atlas plate from C (blue) superimposed. Registration of more than one section to atlas (situation 3, red dashed line) results in repositioning of the other sections in the series relative to the reference atlas. Iterative manual registration of sections is performed until a satisfactory result is reached across the series of images.
(TIF)

S1 File QuickNII user guide. The user guide summarizes the main steps in the anchoring procedure. Detailed and updated information about the user interface and the software functionalities can be found on NITRC.org.
(PDF)
