# Supplementary figures and images for: Spatial registration of serial microscopic brain images to three-dimensional reference atlases with the QuickNII tool
Source: PLoS One. 2019 May 29;14(5):e0216796. doi: 10.1371/journal.pone.0216796 (PMC6541252; doi:10.1371/journal.pone.0216796)

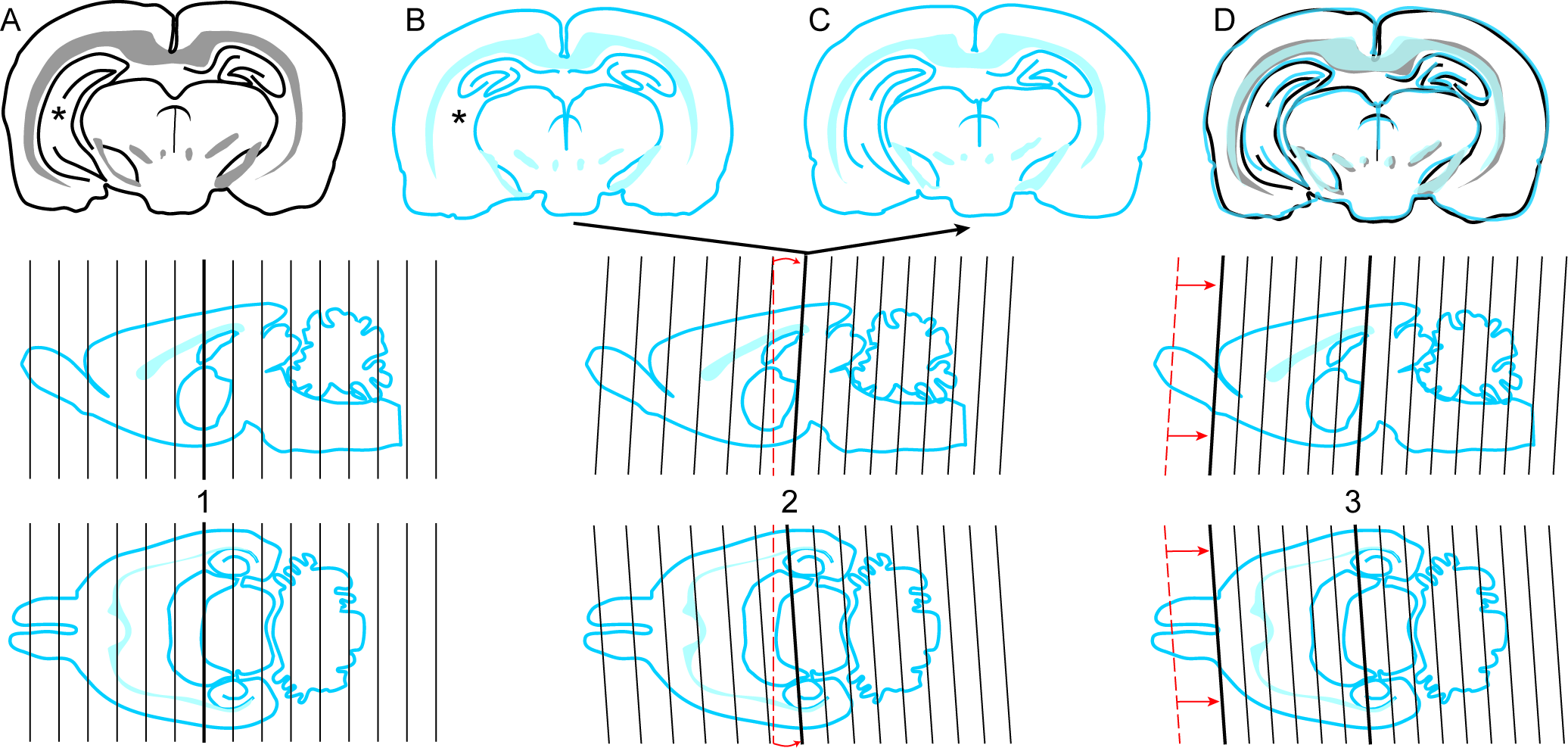

Supplement: S1 Fig — (A) Line drawing of an experimental rat brain section cut at an angle slightly tilted compared to the standard coronal plane. The section is one in a series of sections through the brain. Through the registration process in QuickNII, correctly angled and positioned atlas plates from a 3D rat brain reference atlas (Waxholm Space rat brain atlas) will be superimposed onto each section in the series. (B) Line drawing of a coronal reference atlas plate, found to be close to the section shown in A. The slight difference in angle of orientation between the images in A and B is most easily observed in the hippocampal region, marked with an asterisk. (C) Line drawing of a reference atlas plate matching the location and tilted to fit the angle of orientation of the section shown in A. In the middle and bottom rows, the assumed position of the section shown as is indicated in situation 1 (solid line relative to sagittal and horizontal atlas diagrams, shown in the middle and lower rows, respectively), whereas the angle of orientation of the atlas plate shown in C is indicated in situation 2. The angle of orientation and the scaling (not shown) defined for the adjusted atlas plate is automatically propagated across the series, while distances among sections are automatically stretched or compressed. (D) Section from A (grey) and atlas plate from C (blue) superimposed. Registration of more than one section to atlas (situation 3, red dashed line) results in repositioning of the other sections in the series relative to the reference atlas. Iterative manual registration of sections is performed until a satisfactory result is reached across the series of images. (TIF) [file pone.0216796.s001.tif]
