# Supplementary material for: Spatial registration of serial microscopic brain images to three-dimensional reference atlases with the QuickNII tool
Source: PLoS One. 2019 May 29;14(5):e0216796. doi: 10.1371/journal.pone.0216796 (PMC6541252; doi:10.1371/journal.pone.0216796)
Supplement: S1 File — The user guide summarizes the main steps in the anchoring procedure. Detailed and updated information about the user interface and the software functionalities can be found on NITRC.org. (PDF) [file pone.0216796.s002.pdf]

QuickNII Workflow/User guide

Contents

QuickNII Workflow/User guide 1
1. Introduction....1
1.1 Background 1
1.2 Condition of use....2
1.3 Contact 2
2. Image requirements and pre-processing steps 2
3. Generate your images descriptor file with FileBuider....2
4. Open QuickNII and load the data 3
5. Use landmarks in the images to find their approximate anteroposterior position 5
6. Determine the sectioning angles....7
7. Final adjustments of in plane positions 9
8. Saving results and validation....10
9. Atlas References....11

1. Introduction

1.1 Background

QuickNII is one of several tools developed by the Human Brain Project (HBP) with the aim of facilitating brain atlas based analysis and integration of experimental data and knowledge about the human and rodent brain. QuickNII is a stand-alone tool for user guided affine spatial registration (anchoring) of sectional image data, typically high resolution microscopic images, to a 3D reference atlas space. A key feature in the tool is the capability to generate user defined cut planes through the atlas templates, matching the orientation of the cut plane of the 2D experimental image data. The reference atlas is transformed to match anatomical landmarks in the corresponding experimental images. In this way, the spatial relationship between experimental image and atlas is defined, without introducing transformations in the original experimental images. Following anchoring of a limited number of sections containing key landmarks, transformations are propagated across the entire series of images to reduce the amount of manual steps required.

1.2 Condition of use

QuickNII v1.0 is developed by the Neural Systems Laboratory, Institute of Basic Medical Sciences, University of Oslo, Norway, with support from the European Union Seventh Framework Programme (FP7/2007-2013) under grant agreement no. 604102 (HumanBrain Project).

QuickNII v2.0 is developed by the Neural Systems Laboratory, Institute of Basic Medical Sciences, University of Oslo, Norway, with support from the European Union's Horizon 2020 Framework Programme for Research and Innovation under the Framework Partnership Agreement No. 650003 (HBP FPA).

License: Creative Commons Attribution-NonCommercial-ShareAlike 4.0 International.

Citations: Found on https://www.nitrc.org/projects/quicknii

Download: https://www.nitrc.org/projects/quicknii

1.3 Contact

For technical inquiries and user support contact: Prof. Jan. Bjaalie, j.g.bjaalie@medisin.uio.no

2. Image requirements and pre-processing steps

QuickNII v1.0 and v2.0 supports standard web-compatible image formats, 24-bit PNG and JPEG. Images can be loaded up to the resolution of 16 megapixels (e.g. 4000x4000 or 5000x3000 pixels), however QuickNII does not benefit from image resolutions exceeding the resolution of the monitor in use. For a standard FullHD or WUXGA display (1920x1080 or 1920x1200 pixels) the useful image area is approximately 1500x1000 pixels, using a similar resolution ensures optimal image-loading performance and also eliminates excess storage size.

Preprocessing of images (downsampling, rotation, renaming) can be achieved with other open access software tools (e.g. ImageMagick, Matlab scripts) or python scripts found in many open source libraries (e.g. PIL). Serial section images should be assigned consecutive serial numbers, preferably indicated by three-digit numbers at the end of the file name, e.g. Sample_ID_s001.tif. Section sampling is given by the serial numbers. The section images are collected in a folder. As fulfilling these requirements usually results in preprocessing images (converting to PNG or JPEG and downscaling to screen-like size), QuickNII keeps track of original image dimensions as part of its series descriptor.

3. Generate your images descriptor file with FileBuider

Serial section images should be assigned consecutive serial numbers, preferably indicated by three-digit numbers at the end of the file name, e.g. Sample_ID_s001.tif. Section sampling is given by the serial numbers. The section images are collected in a folder.

Use the small program “FileBuilder.bat” provided with QuickNII. A new window will open, and ask for the folder where your images are located. Point to the correct folder, mark all image files, and click ok.

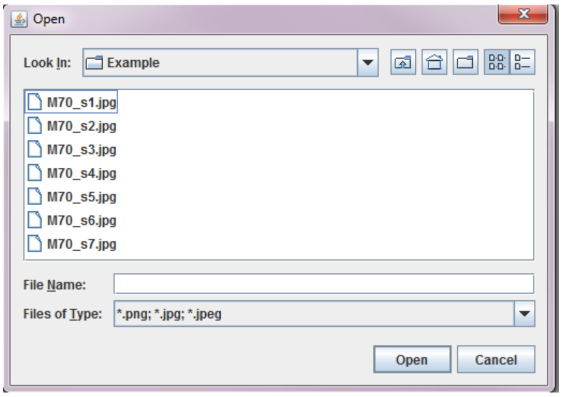

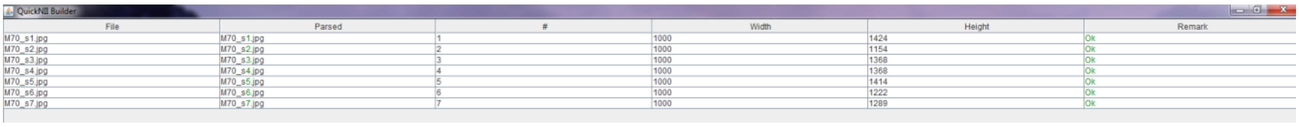

File	Parsed	#	Width	Height	Remark
M70_s1.jpg	M70_s1.jpg	1	1000	1424	Ok
M70_s2.jpg	M70_s2.jpg	2	1000	1154	Ok
M70_s3.jpg	M70_s3.jpg	3	1000	1368	Ok
M70_s4.jpg	M70_s4.jpg	4	1000	1368	Ok
M70_s5.jpg	M70_s5.jpg	5	1000	1414	Ok
M70_s6.jpg	M70_s6.jpg	6	1000	1222	Ok
M70_s7.jpg	M70_s7.jpg	7	1000	1289	Ok

Files will be reviewed and an xml file will be generated. Click “Save xml”. You can now open the dataset in QuickNII. If the section number is not recognized, you have the option to number the images in the file builder.

4. Open QuickNII and load the data

<u>There are two versions of QuickNII for the rodent brains:</u>

- For the mouse brain, the current version is called “QuickNII-ABAv3-pMRI” and contains the Mouse reference atlas from Allen Institute version 3[1,2,].
- For the rat brain, the current version is called “QuickNII-WHSv2” and contains the Waxholm rat reference atlas version 2[3,4].

Open the QuickNII program from the .exe file. Once the program opens, click the **Manage data button**.

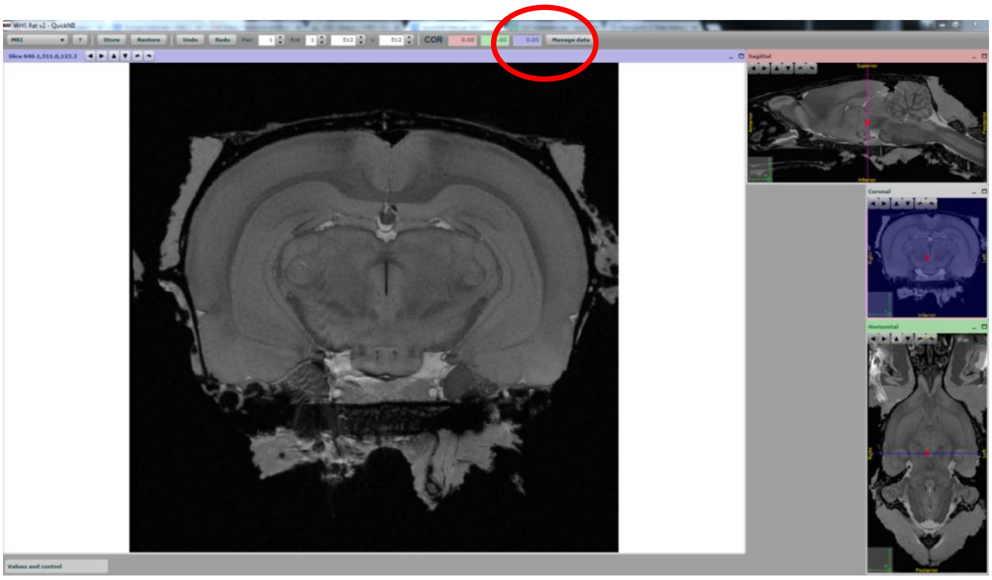

A second window, the data management window, will open. Here you can load your data by clicking the Load button and choosing the .xml file related to your images, choose the “orig file” the first time

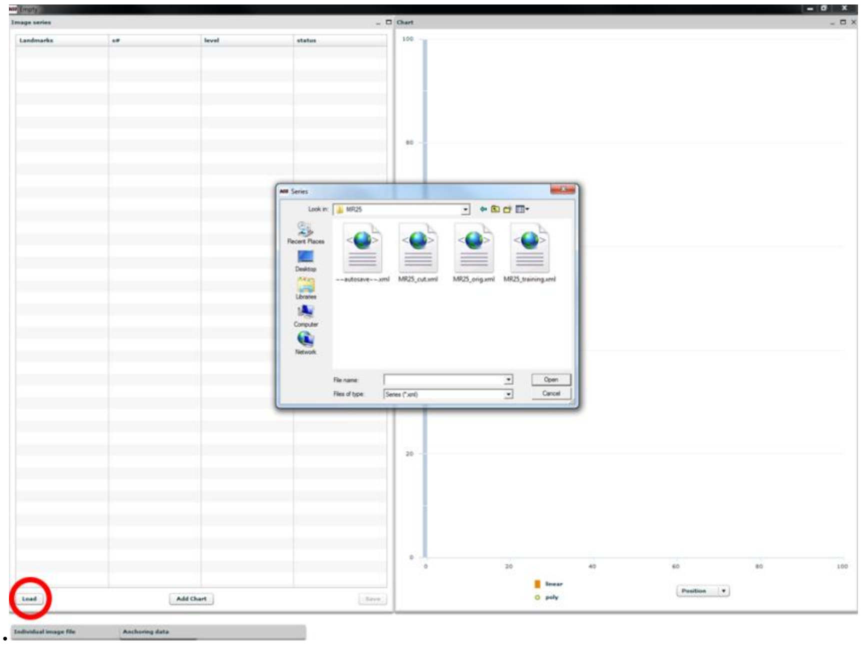

NB! Low resolution .png images must be in the same folder as the .xml files for QuickNII to be able to open the data set. Navigate between the two interfaces by clicking the **Manage data button**. Select sections to work on by using the arrows in the upper right panel or by double-clicking the section number in the data management window.

5. Use landmarks in the images to find their approximate anteroposterior position

The first step in a successful anchoring is to find the approximate anteroposterior position of the slices (Y position for coronal sections). Do this firstly for the first and last section of the series (or first and last sections with clear landmarks). Select sections to work on by using the arrows in the upper right panel or by double-clicking the section number in the data management window.

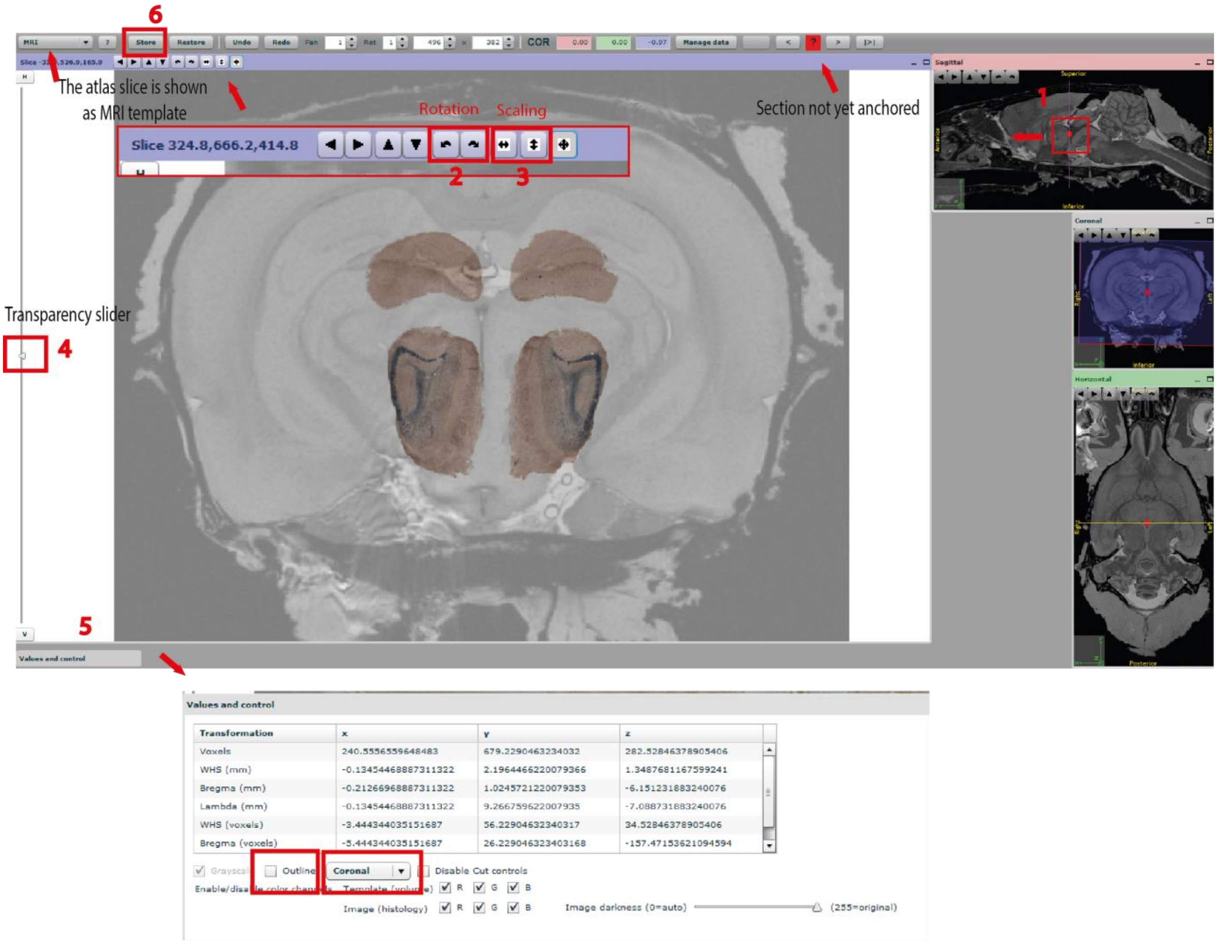

The anteroposterior position is adjusted by clicking and sliding the red circle in the sagittal navigation window (1). After finding the approximate position of your section, determine whether the midline of the section is completely vertical. If not, the rotation of the template can be adjusted using the rotate left/right-buttons (2). The atlas proportions might need adjustment to fit the section. This is done separately for the horizontal and vertical direction by using the scaling buttons (3). **In order to scale** your atlas, press the **space bar** while holding the mouse pointer over the place you want the reference point for scaling. A small

cross will appear. Usually it is easier to choose a side and not place the cross in the middle of the section.

Then, click on the **scaling button**, a double arrow will appear. Then, place your **mouse pointer** at the opposite side of the double cross and press the left button of your mouse and maintain it pressed. While keeping the left button of the mouse pressed down you can now gently drag the atlas in the direction indicated by the double arrow.

To drag in the other direction, choose the other arrow. **The transparency slider** (4) can be used continuously to determine how well the atlas fits the section. By clicking on the **"Values and control"** (5) button in the bottom left corner, choose the section orientation (coronal, sagittal or horizontal). The "outline" button allows you to shift between an outline view or a color view of the atlas segmentations. Save the anchoring by clicking the **Store button** (6) in the upper left panel and a green exclamation mark appears in the upper right panel.

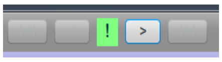

By clicking the roll-down bar in the top left corner, MRI, DTI (for WHS atlas only) or atlas templates can be chosen. It is useful to use these different atlases actively as they give different information that can be used when anchoring. Detailed description of the UI can be found at https://www.nitrc.org/projects/quicknii.

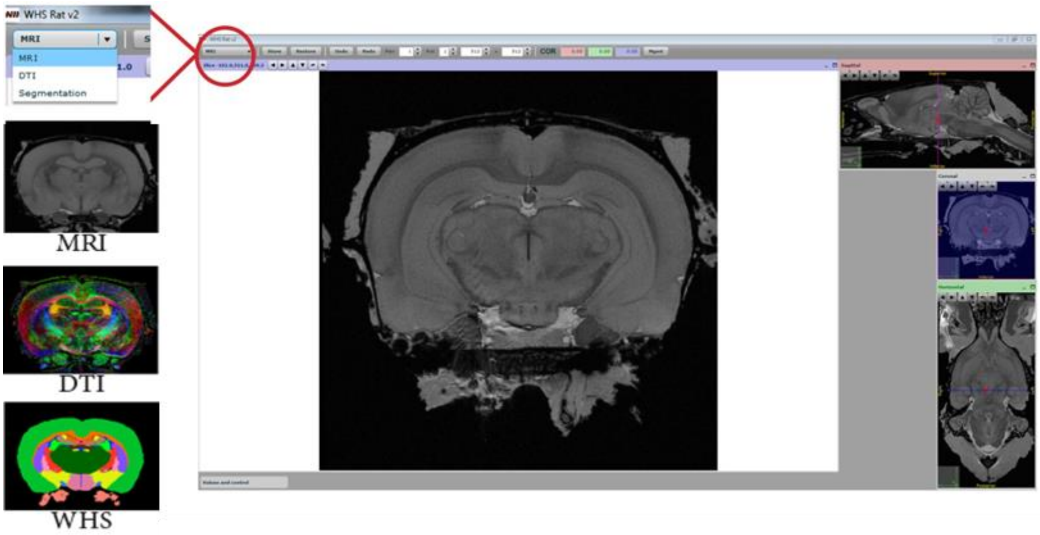

Sections containing key anatomical landmarks are the easiest ones for which to find the anteroposterior position. Some examples are found below:

- ***Genu of the corpus callosum***

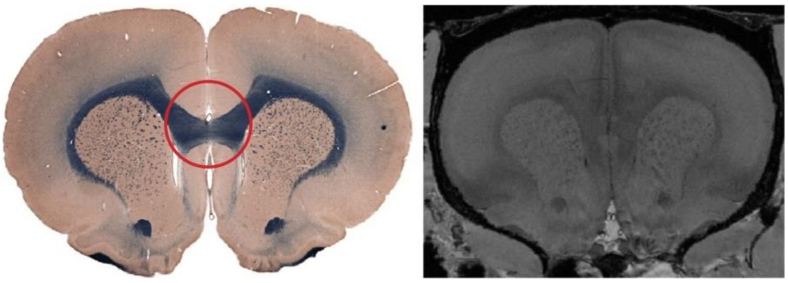

- ***Decussation of the anterior commissure***

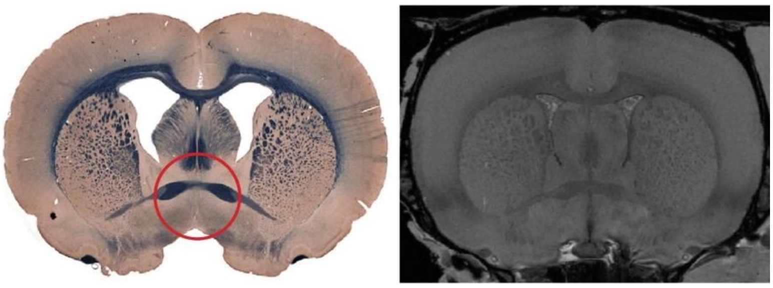

- ***Optic tract in red, mid-level of the anterior hippocampus in green***

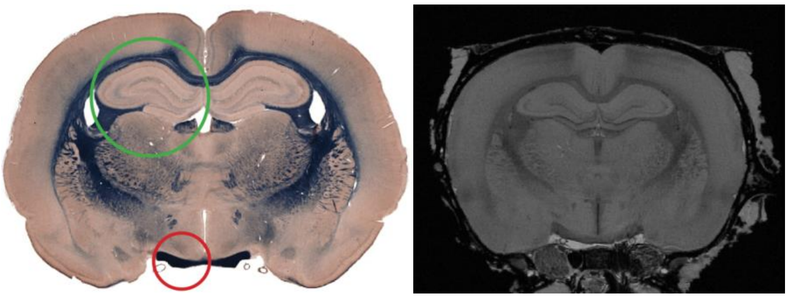

6. Determine the sectioning angles

Next, adjust the angles of the atlas slice to match the angles of sectioning. Even the best sectioning routines can induce small deviations from the vertical and horizontal planes. Furthermore, those angles can vary in a whole series, especially if the tissue was cut into two separate blocks and need to be adjusted. The angles of the atlas need to be adjusted to fit the mediolateral and dorsoventral angles of the sections. This is done in the horizontal and sagittal navigation windows, respectively. Use either the rotation buttons (1) or rotation

handles (2) to tilt the MRI template in the direction needed. Adjust the anteroposterior position to compensate for the rotation.

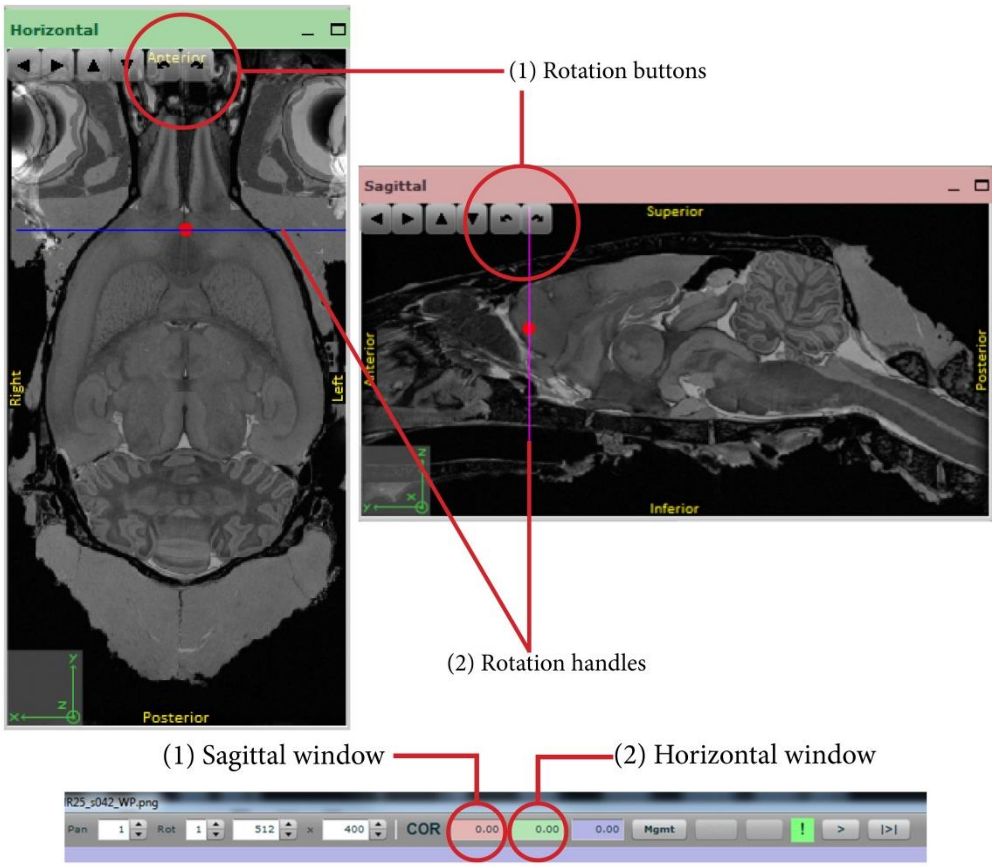

The angles of the current atlas slice relative to the default atlas plane can be read out in the boxes shown above, corresponding to the sagittal (1) and horizontal (2) navigation windows.

In coronal sections, the dorsoventral angle can be determined by examining the relationship between landmarks in dorsal and ventral parts of a section, e.g. between the corpus callosum and anterior commissure, between the dorsal and ventral hippocampus, or between the pons and inferior colliculus. The mediolateral angle can be seen by comparing landmark structures across hemispheres. It is most easily found by examining the development of the corpus callosum, anterior striatum, anterior commissure, anterior hippocampus, or size differences of the cortex in the posterior part of the brain.
Note that the result might look similar with angles that deviate 180 degrees (corresponding to looking at the animal from the back or from the front). To ease the curation process, however, we recommend using the smallest angle possible.

7. Final adjustments of in plane positions

Go now to another image further away and repeat the anchoring procedure. Once the second image is stored, the QuickNII tool will automatically calculate the anteroposterior positions of all the images, as well as propagating the registered angles and scaling. This accelerates the anchoring procedure, i.e. the images will better match the template in an incremented way along with the anchoring of images from your dataset. However, there might be cases where the automatically propagated parameters do not fit the section, for example if a section has been tilted during mounting.

Once defined, apply the same angles to all the sections in the series and save your results!

NB! Every section in the series should be inspected to verify the proposed anchoring, making manual adjustments when necessary.

In-plane rotations are done using buttons in the main window. Rotations in the small coronal window will result in a rotation around the anteroposterior axis.

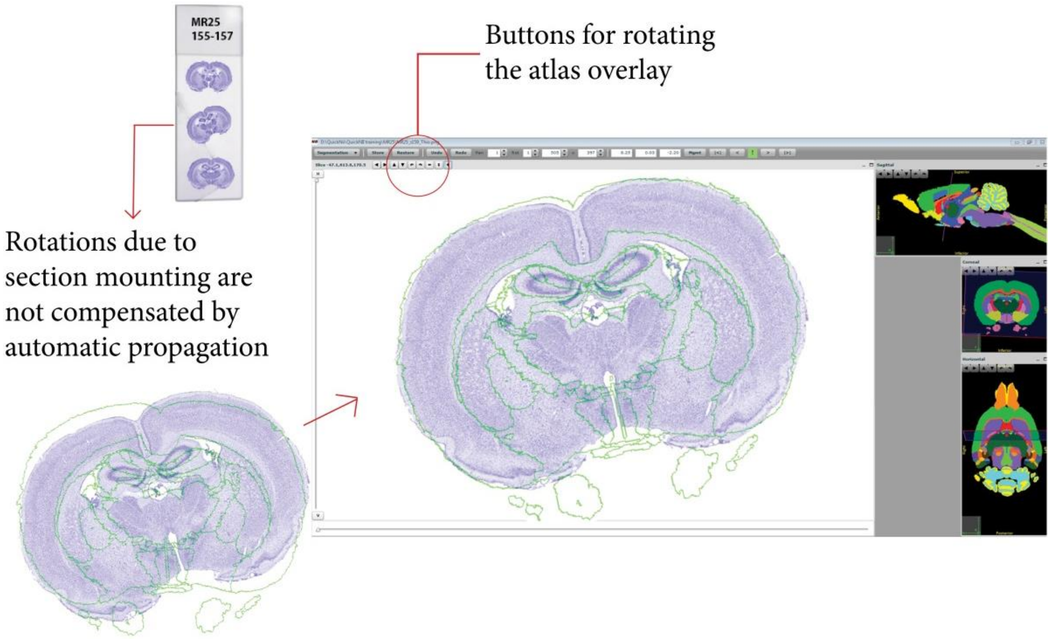

8. Saving results and validation

Remember to save the anchoring result by clicking **"store"**. Export the data by clicking **"Export Propagation"**. A new window will open and you will be able to export results into a new xml file. Type a new name, e.g. initials and date.

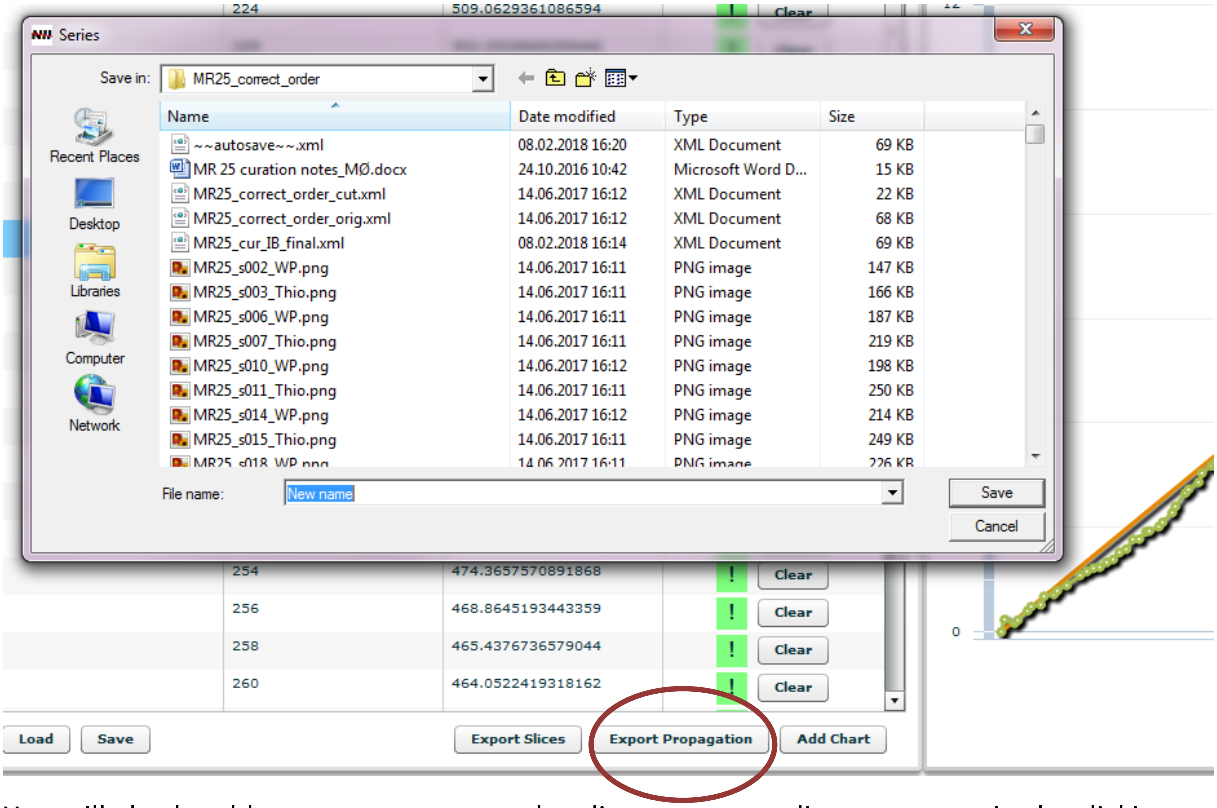

You will also be able to export custom atlas slices corresponding to your series by clicking on the **"export Slices"** button.

Graphs provide an initial indication of registration accuracy. If deviations from the linear regression line are present, a revision of the anchoring should be done. Independent validation by a curator is recommended.

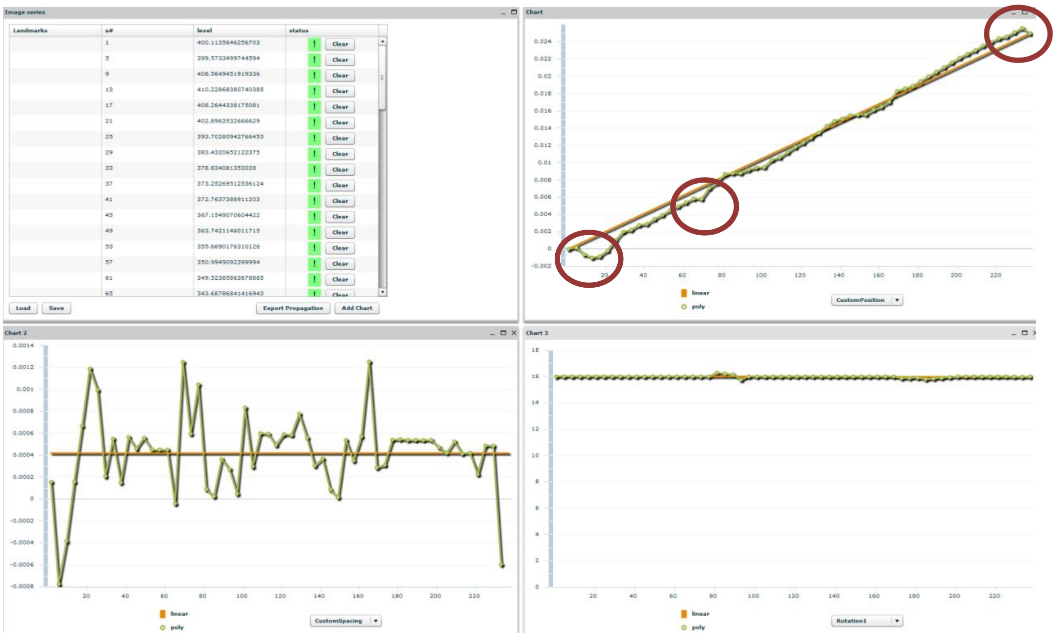

9. Atlas References

1) Lein et al. (2007) Nature 445(7124):168-76
2) Oh, S. W. et al. (2014).A mesoscale connectome of the mouse brain. Nature 508, 207-214, doi:10.1038/nature13186
3) Papp EA et al. (2014). NeuroImage, 97:374-86
4) Kjonigsen et al. (2015) NeuroImage, 108 :441-449
